# Redefining Strategies to Introduce Tolerance-Inducing Cellular Therapy in Human beings to Combat Autoimmunity and Transplantation Reactions

**DOI:** 10.3389/fimmu.2014.00392

**Published:** 2014-08-15

**Authors:** Anja ten Brinke, Irma Joosten, S. Marieke van Ham, Cees van Kooten, Berent Jan Prakken

**Affiliations:** ^1^Department of Immunopathology, Division Research and Landsteiner Laboratory, Sanquin Blood Supply, Academic Medical Center, University of Amsterdam, Amsterdam, Netherlands; ^2^Laboratory for Medical Immunology, Department of Laboratory Medicine, Radboud University Medical Center, Nijmegen, Netherlands; ^3^Department of Nephrology, Leiden University Medical Center, Leiden, Netherlands; ^4^Laboratory for Translational Immunology, Center for Molecular and Cellular Immunology, Wilhelmina Children’s Hospital, University Medical Centre Utrecht, Utrecht, Netherlands

**Keywords:** tolerance, regulatory T cells, dendritic cells, regulatory agencies, autoimmunity, transplantation

## Abstract

Clinical translation of tolerance-inducing cell therapies requires a novel approach focused on innovative networks, patient involvement, and, foremost, a fundamental paradigm shift in thinking from both Academia, and Industry and Regulatory Agencies. Tolerance-inducing cell products differ essentially from conventional drugs. They are personalized and target interactive immunological networks to shift the balance toward tolerance. The human cell products are often absent or fundamentally different in animals. This creates important limitations of pre-clinical animal testing for safety and efficacy of these products and calls for novel translational approaches, which require the combined efforts of the different parties involved. Dedicated international and multidisciplinary consortia that focus on clinical translation are of utmost importance. They can help in informing and educating regulatory policy makers on the unique requirements for these cell products, ranging from pre-clinical studies in animals to *in vitro* human studies. In addition, they can promote reliable immunomonitoring tools. The development of tolerance-inducing cell products requires not only bench-to-bedside but also reverse translation, from bedside back to the bench.

Tolerance-inducing cellular therapies hold great promise for the treatment of patients with chronic inflammation, as seen in autoimmune diseases, and for the prevention of graft rejection and graft-versus-host-disease (GvHD) following transplantation (1, 2). Such treatments possess the key for a true restoration of the immune balance and thus may prevent or minimize the life-long use of immunosuppressive drugs and therewith associated adverse side-effects. Their potential is supported by a vast amount of data from *in vitro* human assays and experimental models (3–7). The first cautious steps to clinical translation have been undertaken by phase I and II studies with both regulatory T-cells (Tregs) and tolerogenic dendritic cells (tolDC) in GvHD, organ transplantation, type I diabetes, and RA (2, 8–11). It is becoming increasingly clear, however, that the intrinsic nature of tolerance-inducing cellular products leads to unique requirements for safe and efficient clinical translation. Here, we highlight some of the most notable hurdles in translation of tolerance-inducing cellular therapies and define some prerequisites that may substantially move the field forward.

## Hurdles in Clinical Translation

**Figure 1:**
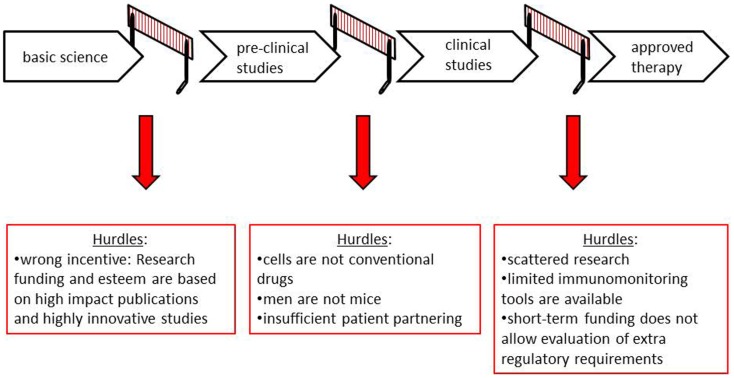
**Steps and hurdles in clinical translation of tolerance-inducing cellular therapies**.

To improve human health using cellular therapies, basic scientific discoveries must be translated into pre-clinical studies [e.g., *in vitro* or *in vivo* (animal) model systems] and subsequently be applied in human subjects in clinical studies. This process entails a number of essential and intricate steps, which require optimal fine-tuning. These steps serve to demonstrate proof-of-concept for the therapeutic potential of the product and to gain optimal information on the safety of the product. Failures or hurdles at any of these steps will substantially slow down the translation of the tolerance-inducing cell therapies (Figure 1 and Table 1).

**Table 1:** **How to move forward**.

Hurdles	How to move forward
Wrong incentive: human studies are less attractive because of academic reward structure	Educate journals, granting organizations, and academic evaluators by creating awareness about the limitations of animal models and value of studies involving human materials
	Promote long-term, supervised grant structure for collaborative research focused on human translational studies
Confused regulators: cellular therapies are treated similarly as conventional drug	Create awareness that cellular therapies need new evaluation tools
Men are not mice: problems cured in animals do not necessarily translate to therapy for human beings	Create awareness about differences between human beings and mice
	Educate about the limitations and added value of mice models
	Use the human model: invest in monitoring of clinical studies
Scattered research by many different research groups	Promote extensive international collaborative networking
Insufficient patient partnering	Involve patients/patients’ organizations more closely in the development of cellular therapies

### Wrong incentives

Publications in high impact journals enhance the career of young academic scientist and their esteem and improve their chances for subsequent funding. Currently, these journals have a preference for highly innovative studies, which are by nature often fundamental. Studies in human subjects are less straightforward and relatively seldom make it to the top journals in the field. Thus, the current academic reward structure has created a wrong incentive for researchers, namely, to keep their research focused on what yields the highest chance of a next top publication and not on what directly or indirectly might be beneficial for translation into human application. This can be considered as a detrimental development for the design of novel clinical therapies.

### Confused regulators

Currently, regulatory bodies treat cellular therapy in a similar fashion as conventional drugs. Unfamiliarity with tolerance-inducing cell therapies is at the basis of this view and is readily explained by the novelty of these therapies. All cell-based therapeutic products (irrespective whether they aim to induce immunological tolerance or immune activation) are essentially different from conventional chemical drugs. Still, for efficacy and safety of cell products, regulatory agencies mostly rely on the models used for testing conventional drugs. This leads to the request for similar product requirements in extensive animal studies on dosage, conventional toxicology, and pharmacodynamics. Many of the characteristics of chemical compounds (solubility, diffusion capacity, turnover into toxic side products) that are tested in such studies, however, do not apply to cells. Therefore, these studies are often of little use for predicting efficacy or safety of a cellular product upon infusion. These studies create additional costs, slow down clinical translation, and hamper effective progression of cell-based therapies.

### Men are not mice

Up to today, much emphasis has been put on the use of experimental animal models as a necessary step toward the clinic, not in the least by regulatory bodies. The use of animal models to learn about mechanisms of action, define therapeutic targets, and design potential therapeutics is beyond doubt. Meta-analysis has shown that only about a third of highly cited animal research is translated at the level of human randomized trials (12). This is in part due to the fact that studies on animals do not always predict the effects of immunomodulatory therapies in human beings correctly. Specifically, for tolerance-inducing therapies, its use needs redefining.

It is important to distinguish the value of animal models based on the type of immunomodulatory therapy used, i.e., biologicals versus cell-based products. Studies in mice have contributed to the success of biologicals, e.g., TNFα blocking therapies. Initial interesting observations that TNFα blockage diminished IL-1 production in *in vitro* human rheumatoid synovial cultures led to extensive studies in mouse models for rheumatoid arthritis, which have resulted into very successful treatment of rheumatoid arthritis with TNFα blocking agents in human beings [as reviewed in Ref. (13)]. Also studies in mice supported evidence that inhibitory molecules like CTLA-4 are involved in immune control and facilitated the use of anti-CTLA-4 antibodies in clinical practice to treat cancer (14).

Clear differences exist between biologicals and cell-based products. First, biologicals are generated in batches, while cell-based products are personalized and often consist of modified cells of individual patients. This obviously complicates animal testing of the latter. Biologicals are also different to cell-based products in terms of mechanism of action. Whereas biologicals initially target one single molecule, cellular products target multiple effector molecules and cells that act in highly interactive networks to shift the immunological balance toward immune activation (anti-cancer therapy) or toward immune tolerance. This fact has important consequences when realizing that the human immune system differs more from rodent models than often is assumed in genetic make-up and immunological maturity. A recent study clearly underscored that genome-wide inflammatory responses in some major diseases/disease models significantly differed between human beings and mice (15). As a consequence, experimental models – though useful in many other ways – mimic poorly human disease. Also for biologicals, the different genetic make-up of the target of the biological in animal models compared to human beings has created a false sense of safety in the past. The development of anti-CD40 ligand antibodies to prevent transplant rejection and treat SLE was halted when the first studies in human beings showed thrombo-embolic complications (16, 17). Subsequent studies indicate that this is caused by the activation of surface FcγRIIa on human platelets, an FcR variant not present on mice platelets (18). Next to genetic differences, also dissimilar maturation of the immune systems in human beings and caged animals may play a role. Mice are mostly studied at young age and are not continuously exposed to pathogens. Consequently, they are more immunological naïve compared to adult patient populations. This further limits the suitability of mouse models as the crucial preparatory step for bringing immunotherapy to the clinic; an experienced and environmentally educated immune system will most certainly react differently to immune interventions. Since cell-based products act on an interactive network of immunological targets, described differences in genetic make-up and immunological maturity make especially evaluation of cell-based products in animal models difficult, and may even create a false sense of safety of the product. Furthermore, inadvertently the preferred choice of administration routes often differs in mice versus men, again questioning if findings can be translated from mouse studies to application in human beings.

The application of tolerance-inducing cellular therapy for autoimmunity is different compared to cellular therapy for other indications. While anti-cancer cellular therapy treats terminally ill patients, without alternative treatment options, tolerance-inducing cellular products target patients with chronic diseases, which are not directly life threatening. Also, tolerance-inducing cells theoretically could even become pro-inflammatory in the pro-inflammatory environment of autoimmune inflammation. This asks for additional safety precautions and additional forms of experimental testing. Some human cell types, however, are non-existent in animals or show great differences in molecular phenotype and function. For instance, human- and mouse-induced Treg have different regulators of their transcription and show different stability (19, 20). This may lead to wrong leads and clues for development of novel therapies in human beings.

Interestingly enough, the absence of appropriate small animal models has not hampered the HIV research field in making tremendous advances. On the contrary, it may even have stimulated a more rapid translation from bench-to-bedside.

## How to Move Forward

We foresee several ways to speed up the translation of tolerance-inducing cell therapies into the clinic, as discussed below.

### Change incentives

The changed environment requires a transformation in the appraisal of science. Because of the limitations of animal studies for the final translation toward human beings, academic evaluators and journals need to re-evaluate the value of the complex and often more messy studies in human beings. Likewise, as single research groups cannot perform such extensive human studies, granting organizations should give priority support to large international and multidisciplinary consortia to prevent overlap and to accelerate the process.

### Educate regulators

Therapeutic cells have different mechanisms of action and a different safety profile compared to conventional chemical drugs or “biologicals” and thus need to fit different regulatory requirements. Currently, awareness is slowly arising that such therapies require new requirements and evaluative tools (21). It is imperative that academia, industry, and regulators work together to adapt the regulatory policy to meet these requirements. Initiatives such as from Burroughs Welcome Fund (BWF) (22), which funds academic research on innovative approaches for assessing the safety and efficacy of new therapies, and as a result, promotes regulatory policy decisions based on the state-of-the-art science, are a promising start.

### Use a model as a model

It is clear that men are not mice. If we want to cure a human disease, we should keep this as a primary focus when considering the use of experimental models. Though seemingly obvious, it requires a paradigm shift in thinking, not only from researchers but also from journals and regulators. To date, major journals and regulators almost always request validation in animal models as a golden standard. We believe that for the development of cellular therapy this is not the right approach. Even “humanized” mouse models can only mimic the human system and never completely reflect a human disease with its enormous heterogeneity and complexity (23). Animal models should be used for what they are developed for, namely, as model for mechanistic pathways and not as model for a specific disease. As such, they can be instrumental for specific issues that cannot be addressed in human beings directly, like the *in vivo* interaction of cellular products with other immune cells and local tissues and effects thereof. Animal models may be specifically valuable to evaluate issues as homing, longevity, and stability of the tolerance-inducing cellular products. Still, definite proof for these issues needs to be obtained in clinical trials in human beings. Therefore, while being a valuable source for mechanistic data and safety data on product stability, animal models should not be used as a single decisive requirement for tolerance-inducing cellular therapy development.

### Use the human model

Efforts should be made to obtain data from human studies. For example, migration potential, dosing, and life-span of tolerance-inducing cell therapies can be deduced from experiences with cellular therapy in cancer. Sometimes, a case-report in human beings can be as valuable and informative as a study in a mouse model (24). Certainly, the first trial on tolerance-inducing cell therapies will yield valuable information. Phase “0” studies using very low dosage of the product may be specifically considered to establish the stability of the tolerance-promoting capacity of the cellular product. Finally, to establish clinical efficacy of novel tolerance-inducing cell therapies, it is imperative to define reliable surrogate outcome parameters that reflect effective immunotherapy. This needs to arise from reverse translation, i.e., from bedside-to-bench, and thus again through studies in human beings (25). For that reason, we strongly advocate that development of cell-specific immunomonitoring technology in human beings is further advanced and promoted. Thus, clinical and animal model-based observations ideally should complement each other and lead to insights, which would not be possible by either one alone.

### Scientific collaboration

The development of cellular therapies can only be realized through extensive international collaborative networking. Experience and data should be shared to prevent repetition, waste of funding, and to reach consensus on difficult questions. This requires a different mind-set from the general competitive nature of basic biomedical research. Promising initiatives in this respect are the ONE study, a unified approach evaluating cellular immunotherapy in solid organ transplantation, the Immune Tolerance Network (ITN, sponsored by NIAID), the recently initiated EU COST Action A FACTT (Action to Focus and Accelerate tolerance-inducing cellular therapies), and the development of broad platforms for biological studies such as UCAN-U (Understanding Childhood Arthritis Network) (26).

### Involve the stakeholders

Patients (and patient organizations) are the true stakeholders of cellular therapy development. They should be more closely involved in risk and benefit analysis of cellular therapies. There are various examples of patient organizations as true catalysts for novel development, for example, in the field of muscular dystrophy (27). We propose that basic scientists, clinicians, regulators, industry, and patients collaboratively develop guidelines for strategic development of defined cellular therapies.

## Author Contributions

Anja ten Brinke, Irma Joosten, S. Marieke van Ham, Cees van Kooten, and Berent Jan Prakken wrote the paper.

## Conflict of Interest Statement

The authors declare that the research was conducted in the absence of any commercial or financial relationships that could be construed as a potential conflict of interest.
